# Coexistence of non-Hodgkin lymphoma and multisystem hydatid cysts; successful albendazole therapy for muscular hydatid cysts: A case report

**DOI:** 10.1016/j.ijscr.2024.110115

**Published:** 2024-08-06

**Authors:** Zainab Srouji, Zeina Talla, Zain Douba, Wael Alkhaleel, Ahmad Ghazal

**Affiliations:** a Faculty of Medicine, University of Aleppo, Aleppo, Syria; b CME Office, Faculty of Medicine, University of Aleppo, Aleppo, Syria; c Department of Hematology, Syrian Arab Republic Ministry of Health, Aleppo, Syria; d Department of General Surgery, Faculty of Medicine, University of Aleppo, Aleppo, Syria

**Keywords:** Hydatid cyst, Non-Hodgkin lymphoma, Albendazole, Muscles, Case report

## Abstract

**Introduction:**

“Hydatid cyst” or cystic Echinococcosis is a parasitic infection caused by the larval stage of Echinococcus granulosus. The liver and lungs are the most common sites to occur. Incidence in muscles is exceptionally rare. Surgery has been the traditional approach for treatment of cystic echinococcusis.

**Presentation of case:**

We report a rare case of 44 years old man with multiple hydatid cysts; liver, lungs, paraspinal muscles. The muscular cyst had manifested as a swelling in his back and was the principal clinical presentation as it caused pain and discomfort.

He was treated with Albendazole, and a thoracic surgery for the management of the lung cysts had been performed. On admission and after his surgery, lymphadenopathy had manifested and following adequate diagnostic modalities he was diagnosed with Non-Hodgkin lymphoma. Then, after three months, physical examination revealed significant reduction in the size of his back cyst that was no longer visible.

**Discussion:**

Here we present a successful treatment for muscular hydatid cysts. While prior reports have managed it surgically; albendazole has played a significant role in our case, in addition to the diagnosis of the NHL in the course of managing multiple hydatid cysts.

**Conclusion:**

The presence of non-Hodgkin lymphoma alongside hepatic cystic disease is rare, and the coexistence of NHL and muscular hydatidosis is unprecedented in medical literature.

## Highlights

• Cystic hydatidosis should be considered as a differential diagnosis for any cystic lesion.
• A rare association of NHL and multiple hydatid cysts in one case.
• Successful treatment for muscular hydatid cysts with Albendazole.

## 1 Introduction

Cystic hydatidosis is a zoonotic disease caused by the larvae of echinococcus species, in which humans are infected through the consumption of food and water contaminated with the eggs of parasites of the echinococcus granulosus or through direct contact with the host [1,2].

Although hydatid cyst can occur almost anywhere in the human body, it usually affects the liver (∼70 %) followed by the lungs (∼20 %), whereas musculoskeletal involvement is rare and contributes to (∼0.5 %–5 %) of cases [3].

Hydatid cysts are usually asymptomatic, and the symptoms usually develop depending on the size of the cyst and vary according to its location [1]. Hydatid cysts in the muscles show no characteristic clinical signs and symptoms [4].

The standard therapy of hydatid cysts is surgery while medical treatment plays a more significant role in inoperable patients and multiple organ involvement [5].

Our report handles a case of 44 years old man with multiple hydatid cysts in liver, lungs, paraspinal muscles and Non-Hodgkin lymphoma.

Prior reports on muscular localization of hydatid cysts have managed it principally through various surgical techniques, but our report is the first to mention association between muscular hydatidosis and Non-Hodgkin lymphoma in addition to an expected response for treatment with albendazole.

## 2 Method

This case has been reported in line with the SCARE criteria [6].

## 3 Presentation of case

A 44 years old man presented to our surgical unit with 1-year history of a painful swelling in his back under the left scapula.

His past medical history included cough, viscous bloody sputum, fever and extreme fatigue; the previous symptoms manifested a year ago and lasted for three months and then improved gradually.

He had no surgical or family histories. His appetite was good with minimal weight loss, smoked 35 packs/year and lived in a rural area.

Examination of the mass revealed mild tenderness and fluctuation.

A laboratory examination, including a complete blood cell count, serum chemistries and a coagulation profile showed values within normal ranges.

**Fig. 1:**
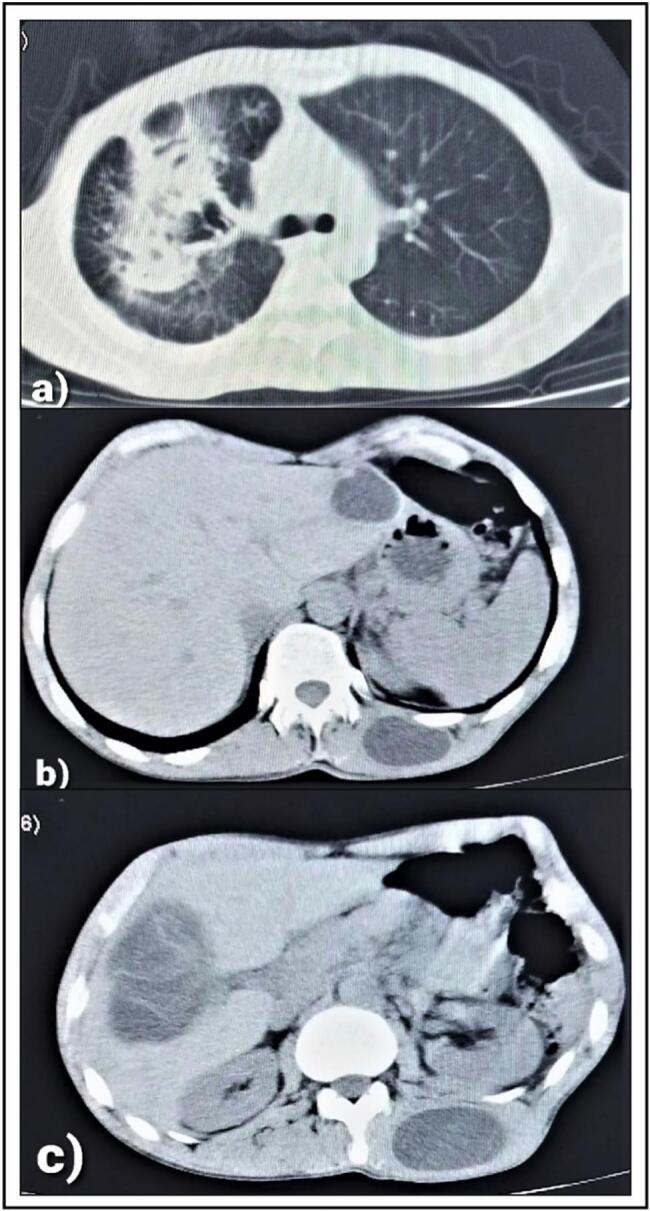
CT scan reveals: (A) two open cysts in the right lung. (B) 4 cm cyst in the left hepatic lobe and the cyst in paraspinal muscles on the left side of the patient. (C) 8 cm cyst in the right hepatic lobe and the cyst in paraspinal muscles on the left side of the patient.

Initially, ultrasound examination of the lump demonstrated cystic formation, smooth edges, turbid content with membranes. The dimensions were 3.5 × 6.5 cm; most likely to be a hydatid cyst. The CT showed two open cysts in the right lung with air-fluid level, two cysts in the liver; 8 cm in the right lobe and 4 cm in the left lobe, in addition to the cyst in the paraspinal muscles on the left side (Fig. 1).

The patient was started on albendazole and it was decided to operate on the lung first. A right intercostal incision was made and two open cysts located in the right upper lobe were found. Their cavities were opened, the germinal membranes were excised, the fistulae were closed using vicryl and the cavities were reclosed.

Following thoracic surgery and during hospitalization period, the patient started complaining about a pain in the groin, palpation revealed lymph nodes enlargements in the inguinal area, the neck and the axilla.

US findings for the lymph nodes were suspicious. An excisional biopsy from the right axilla was taken and the pathology report came back with Non-Hodgkin lymphoma (T cell rich large B cell lymphoma).

Afterwards, contact with the patient was lost for two months for his own circumstances, but during this period, he continued on taking Albendazole. When he returned for follow up, physical examination revealed that the mass on the left side of his back had decreased significantly in size and was barely palpable.

**Fig. 2:**
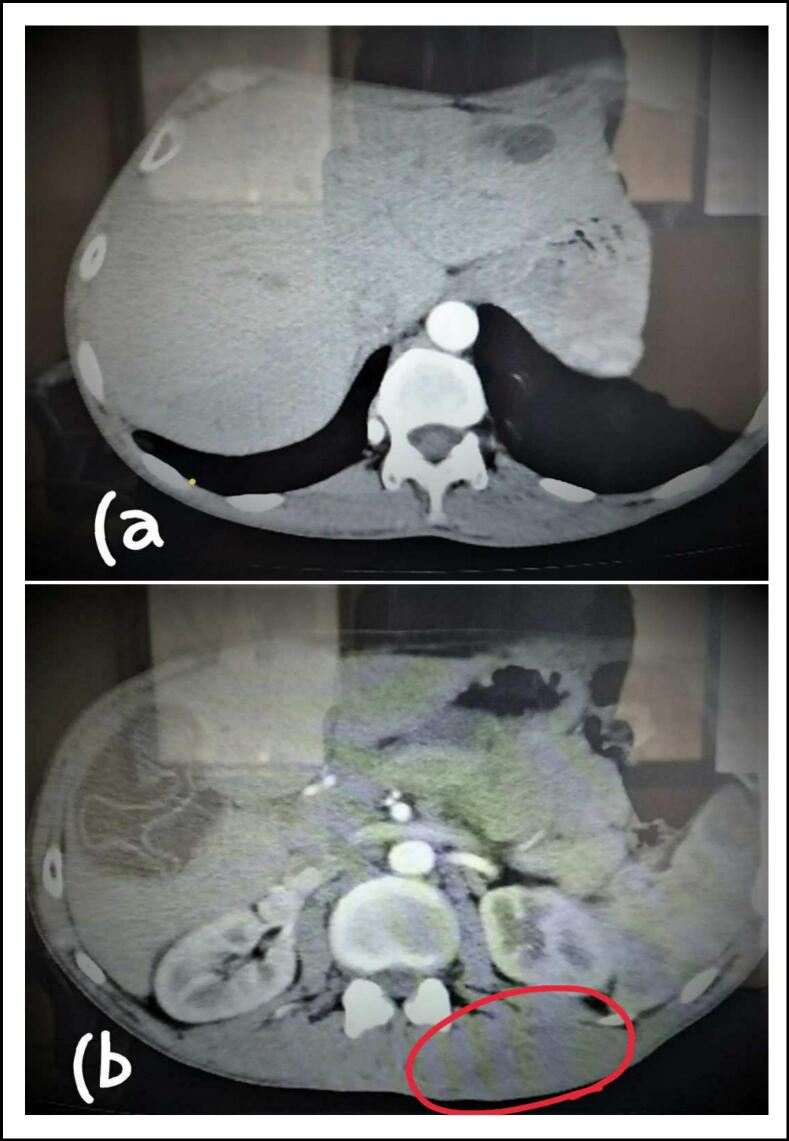
Multislice CT reveals: (A) the cyst in the left lobe that has reduced to 1 cm. (B) The cyst in the right lobe and regression of the cyst in paraspinal muscles on the left side (red circle). (For interpretation of the references to colour in this figure legend, the reader is referred to the web version of this article.)

Multislice CT showed regression of the cyst in the paraspinal muscles on the left side, and the cyst in the left hepatic lobe had reduced in size to 1 cm in diameter with no change in the cyst of the right lobe. These findings came after 3 months of treatment with Albendazole; (one month before surgery and two months after). The CT also revealed lymphadenopathy in the mediastinum; the largest measured 1 cm in diameter, and lymph nodes aggregation in the right and left axillae, the largest were in the left side 16 × 27 mm (Fig. 2).

The oncologist decided to operate on the liver cyst before administration of chemotherapy, laparoscopy was done to remove the cyst in the right hepatic lobe and the patient received postoperative pharmacological therapy with Albendazole. He was then followed up by oncology department and received his chemotherapy.

## 4 Discussion

Echinococcal disease is a worldwide distributed parasitic infection caused by the tapeworm echinococcus with higher incidence rates at endemic regions such as the Mediterranean and the middle east including our country, Syria [7].

The most common sites for cysts to form are the liver and the lungs, and less frequently, the muscles.

When cysts form at the expense of back muscles, pain and discomfort especially while lying down will develop, as in our patient. It could also exhibit compressive neurological symptoms.

Surgery has been the traditional approach for treatment of cystic hydatidosis; chemical therapy may be used for definitive management in selected cases [8]. It is also a useful adjuvant therapy to surgery and percutaneous treatment.

Albendazole is the primary antiparasitic agent for treatment of echinococcus granulosus [9].

Cumulative experience with albendazole suggests that treatment leads to cyst resolution in up to 30 % of patients, size reduction in another 30 % to 50 % and no change in 20 % to 40 % [9].

Frankly, it was decided to operate on the muscular cyst, but due to the fact that the patient lived in a rural distant area and the difficulties in accessing prompt medical help in our afflicted country, his delay came back with benefit as his back cyst doesn't need excision anymore.

The cyst in the left hepatic lobe was not removed due to its small size.

Prior reports on muscular hydatid cysts had managed it mainly surgically. However, to our knowledge, the medical literature contains only one report of a complete cure of muscular hydatidosis only by means of albendazole therapy [10].

Non-Hodgkin lymphomas (NHLs) are tumors originating from lymphoid tissues, mainly of lymph nodes. NHL is the most prevalent hematopoietic neoplasm, representing approximately 4.3 % of all cancer diagnoses. Chemotherapy represents the main treatment.

When lymphadenopathy had manifested after thoracic surgery, it raised the suspicion of an underlying problem, so proper diagnostic modalities were performed which led eventually to the diagnosis of the tumor.

Although coexistence of NHL and hepatic cystic disease has been reported before, association between NHL and muscular hydatidosis has not been reported before.

## 5 Conclusion

Here we report a coexistence of NHL and multiple hydatid cysts in one case. This association is rare, and to our knowledge has not been reported before. In addition to the unprecedented response for albendazole therapy, on the other hand our patient was treated successfully for both conditions without any signs of relapse.

In endemic countries, cystic hydatid disease should be considered as a differential diagnosis for every cystic lesion in any anatomical location [2].

Here we present a successful treatment for multiple hydatid cysts; in which albendazole has played a significant role, in addition to the diagnosis of the lymphoma in the course of managing the parasitic infection.

## Consent for publication

Written informed consent was obtained from the patient for publication of this case report and accompanying images. A copy of the written consent is available for review by the Editor-in-Chief of this journal on request.

## Ethical approval

Ethical approval was waived by the board of Faculty of Medicine at Aleppo University for this type of articles “case report”.

## Funding

None.

## Author contribution

Zainab Srouji: Writing original draft, review and editing, visualization, literature review, and the corresponding author who submitted the paper for publication.

Zeina Talla: Writing, review and editing, visualization, literature review.

Zein Douba: Supervision, validation and reviewing.

Wael Alkhaleel: Conceptualization, investigation, resources and visualization.

Ahmad Ghazal: Performed and supervised the operation, conceptualization and reviewing.

All authors read and approved the final manuscript.

## Guarantor

Zainab Srouji.

## Research registration number

N/A.

## Conflict of interest statement

None.
